# The effectiveness of sit-stand workstations for changing office workers’ sitting time: results from the Stand@Work randomized controlled trial pilot

**DOI:** 10.1186/s12966-014-0127-7

**Published:** 2014-10-08

**Authors:** Josephine Y Chau, Michelle Daley, Scott Dunn, Anu Srinivasan, Anna Do, Adrian E Bauman, Hidde P van der Ploeg

**Affiliations:** Prevention Research Collaboration, Sydney School of Public Health, University of Sydney, 92-94 Parramatta Road, Camperdown, NSW 2050 Australia; Heart Foundation New South Wales, 3/80 William St, Sydney, NSW 2011 Australia; Centre for Epidemiology and Evidence, New South Wales Ministry of Health, Locked Mail Bag 961, North Sydney, NSW 2059 Australia; Department of Public and Occupational Health, EMGO Institute for Health and Care Research, VU University Medical Center, Van der Boechorststraat 7, 1081BT Amsterdam, The Netherlands

**Keywords:** Sedentary behavior, Sit-stand workstation, Workplace, Randomized controlled trial

## Abstract

**Background:**

Prolonged sitting time is detrimental for health. Individuals with desk-based occupations tend to sit a great deal and sit-stand workstations have been identified as a potential strategy to reduce sitting time. Hence, the objective of the current study was to examine the effects of using sit-stand workstations on office workers’ sitting time at work and over the whole day.

**Methods:**

We conducted a randomized controlled trial pilot with crossover design and waiting list control in Sydney, Australia from September 2011 to July 2012 (n = 42; 86% female; mean age 38 ± 11 years). Participants used a sit-stand workstation for four weeks in the intervention condition. In the time-matched control condition, participants received nothing and crossed over to the intervention condition after four weeks. The primary outcomes, sitting, standing and walking time at work, were assessed before and after using the workstations with ActivPALs and self-report questionnaires. Secondary outcomes, domain-specific sitting over the whole day, were assessed by self-report. Linear mixed models estimated changes in outcomes adjusting for measurement time, study grouping and covariates.

**Results:**

Intervention participants significantly reduced objectively assessed time spent sitting at work by 73 min/workday (95% CI: −106,-39) and increased standing time at work by 65 min/workday (95% CI: 47, 83); these changes were significant relative to controls (p = 0.004 and p < 0.001, respectively). Total sitting time significantly declined in intervention participants (−80 min/workday; 95% CI: −155, −4).

**Conclusions:**

This study shows that introducing sit-stand workstations in the office can reduce desk-based workers’ sitting time at work in the short term. Larger scale studies on more representative samples are needed to determine the public health impact of sit-stand workstations.

**Trial registration:**

ACTRN12612000072819

**Electronic supplementary material:**

The online version of this article (doi:10.1186/s12966-014-0127-7) contains supplementary material, which is available to authorized users.

## Introduction

High amounts of time spent in sedentary behaviour have been linked with increased risk for a range of health outcomes including type 2 diabetes, cardiovascular disease, and mortality [1-8]. The prevalence of adults working in jobs that require mostly sitting is high [9], and occupations have become more sedentary and less physically active over the past few decades [10]. Working adults spend an estimated one third to three quarters of their work time sitting [11-14]. Thus, reducing sitting time may have good potential for chronic disease prevention in working populations, with the workplace as an appropriate intervention setting. However, workplace interventions have mainly addressed sedentary behaviour as a secondary outcome to promoting physical activity and there is a need for more trials that specifically target sedentary behaviour [15].

Modifying the occupational environment through the introduction of sit-stand workstations is one potential method for reducing prolonged sitting during the workday among office workers. Sit-stand workstations allow the user to alternate between sitting and standing postures at their desk, giving them the opportunity to reduce their sitting time at work. Evidence about sit-stand workstations from the occupational health and ergonomics literature has concentrated on musculoskeletal health and work performance [16-21]. More recently, public health focused research has examined sit-stand desks as tools for reducing sitting time with the aim of preventing chronic illness [22]. Five recent intervention studies [23-27], and one natural experiment [28] have examined the impact of using sit-stand workstations on desk-based workers’ sitting time in office settings with promising results. Two recent systematic reviews on activity permissive workstations revealed promising results for sit-stand workstations but called for higher quality studies [29,30]. Few trials to date have been conducted using a randomised controlled design [27], and few studies have examined the effects of sit-stand workstations on sitting time outside of work.

This paper describes the results of a pilot randomised controlled trial, the Stand@Work study. The aim of this study was to investigate the effect of using sit-stand workstations on office workers’ sitting time at work and over the whole day.

## Methods

The study was approved by the University of Sydney Human Research Ethics Committee (No. 08-2011/14067) and all participants gave written informed consent. The study is registered with the Australian New Zealand Clinical Trials Registry (No. ACTRN 12612000072819).

### Participants

Participants were staff from a non-government health agency in New South Wales, Australia, aged over 18 years, employed at least three days per week, and who had sufficient English language proficiency to undertake study tasks. The project was advertised to staff as part of their workplace wellness program via internal mail, staff meetings and information fliers in the office. Staff members who were interested in participating, contacted the research team and received additional project information and an expression of interest form. They could then join the study ballot by returning the expression of interest form.

### Design

**Figure 1:**
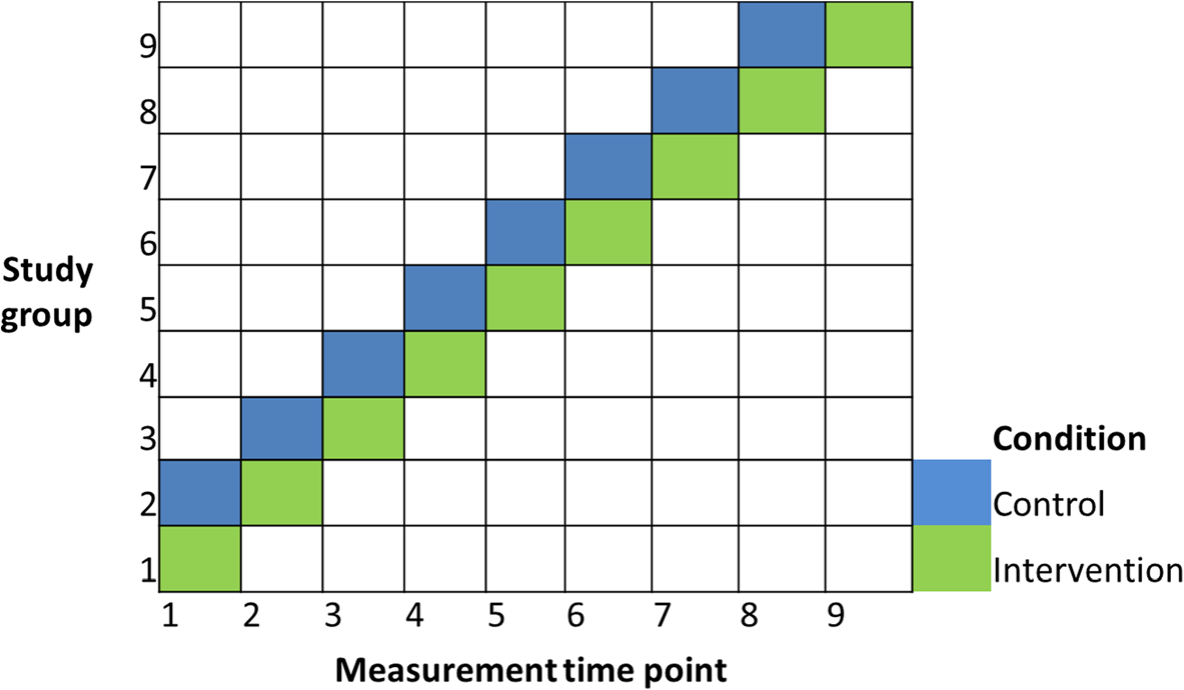
**Design of the Stand@Work study – randomised controlled trial with crossover and waitlist control.**

This pilot study had a randomised controlled trial design with crossover and rolling recruitment and a waitlist control condition (Figure 1). Eligible staff members who returned an expression of interest form were randomly drawn from a ballot by a researcher in the presence of potential participants and other researchers, and were included in the study after providing written informed consent. The first four participants drawn from the ballot were allocated to the intervention group to use a sit-stand workstation for four weeks (Figure 1, study group 1), the next four participants drawn from the ballot served as the control group (Figure 1, study group 2). The remaining participants were assigned to the waitlist control condition and were placed on the waiting list in seven groups (four to five people per group). After the initial four weeks, the previous control group (study group 2) received the intervention with the next group from the ballot draw serving as their controls (study group 3). This was repeated until all nine groups had received the intervention. This study design was used to maximise the evaluation sample size taking into account the five available sit-stand workstations. Allocation concealment was not possible due to the open plan nature of the study office environment. Data collection ran from September 2011 to July 2012.

### Intervention

The intervention involved giving participants a sit-stand workstation (Ergotron Workfit S) to use for four weeks for all workdays (3 to 5 workdays per week depending on full-time or part-time employment status). The sit-stand workstation allowed office workers to vary their posture throughout the workday between sitting and standing. Prior to receiving the sit-stand workstation, participants received brief training on how to use the sit-stand workstation, including how to adjust workstation height and manufacturer specified ergonomic set ups while sitting or standing. Participants also completed an ergonomic self-assessment prior to use and were advised to increase their standing time gradually to prevent injury and fatigue.

### Outcome assessment

Assessments, consisting of objective monitoring and self-report questionnaires, took place at three time points scheduled four weeks apart. Assessment 1 was six weeks pre-intervention, assessment 2 at two weeks pre-intervention and assessment 3 in the third week of the intervention (Figure 1). Changes between assessments 1 and 2 were treated as the control condition, changes between assessments 2 and 3 as the intervention condition. The exception was the first intervention group, which only participated in assessments 2 and 3, because they could not serve as a time synchronized control condition.

During all three assessments participants wore an ActivPAL activity monitor (model ActivPAL3; PAL Technologies Ltd., Glasgow, UK) for the working week during work time and kept a monitoring log book to note the times they wore the ActivPAL each day, the days they worked, and the times they started and finished work on each of those days. We used the ActivPAL to objectively measure time spent sitting, standing and stepping at work. The ActivPAL is a small activity monitor that is worn on the front mid thigh [31,32]. The ActivPAL identifies episodes of sitting/lying, standing or stepping based on the inclination of the thigh. It has demonstrated reliability and validity for measuring postures and activities of daily living (sitting, standing, stepping) [31]: good to excellent interdevice reliability (intraclass correlation coefficients ranged from 0.79 to 0.99); good device-observer agreement for sitting, standing and stepping (mean percentage difference between ActivPAL and observer of 0.19%, −1.4% and −2.0%, respectively, on Bland-Altman plots). ActivPAL has also been shown to be responsive to change [33]. ActivPAL data were first processed using proprietary software (ActivPAL v6.1.2.17, PAL Technologies Ltd., Glasgow, UK) which classified the data into sitting, standing and stepping categories. We further analysed the data using custom software (HSC analysis software v2.19, Philippa Dall and Malcolm Granat, Glasgow Caledonian University) which allowed us to isolate participants’ work time data based on their work start and finish times reported in their monitoring log.

Additionally, participants completed questionnaires at each assessment. We used the Occupational Sitting and Physical Activity Questionnaire (OSPAQ) [34] to measure time spent sitting, standing, walking, and doing more physically demanding tasks at work. It has demonstrated excellent test-retest reliability for assessing sitting, standing, walking and doing physically demanding tasks at work (ICC = 0.54-0.89) [34]. Validity correlations for occupational sitting, standing and walking measures were 0.52, 0.49 and 0.27, respectively, against Actigraph accelerometers [34].

Sitting time over the whole day was measured with the Workforce Sitting Questionnaire (WSQ) [35], which measures total and domain-specific sitting based on work and non-workdays in working adults. The WSQ asked participants to report their time spent sitting (1) while travelling to and from places; (2) while at work; (3) while watching TV; (4) while using a computer at home; and (5) while doing other leisure activities on a workday and a non-workday in the last 7 days. It has acceptable test-retest reliability (ICC = 0.76) and validity against Actigraph accelerometers (r = 0.40) for assessing total sitting time based on work and non-workdays [35]. The WSQ is also a reliable (ICC = 0.63) and valid (r = 0.45) measure of domain-specific sitting at work on a workday [35].

Participants also provided information about their sex, age, height, weight, employment status (full time or part time, number of days worked, and hours worked per week), type of office arrangement (own office or open plan) and highest level of education. Additionally, Body Mass Index (BMI) was calculated as kg.m^−2^.

### Statistical analyses

Statistical analyses were carried out with SAS/STAT software, Version 9.3 of the SAS system for Windows with statistical significance set at p < 0.05 (two-tailed). We used linear mixed models to estimate changes in self-reported and objectively assessed time spent sitting, standing and walking/stepping before and after the use of a sit-stand workstation. We used the SAS procedure, PROC MIXED, to estimate the effect of the intervention, allowing adjustments for measurement time, potential confounders, clustering of participants within groups, and repeated measures on the same participant. The model included the fixed effects of assessment time (assessment 1, assessment 2, assessment 3), as well as the average group measurement time, a blocking variable used to account for the effect of time. Estimates were adjusted for the following potential confounding variables: age, BMI (continuous), office type (open plan or own office), highest level of education completed (high school; trade, technical certificate or diploma; university; or other), and full time or part time work. Participant, nested within group number (order in which group received intervention), was included as a random component to adjust for within-subject correlation. For the control condition, we defined change in outcome as the difference in the adjusted estimates of outcome measures at assessment 1 and assessment 2 (assessment 2 minus 1); for the intervention condition, this change was defined as the difference in the adjusted estimates of outcome measures at assessment 2 and assessment 3 (assessment 3 minus 2). ActivPAL data were considered valid when the participant wore the device for at least 75% of their time at work. Analyses of ActivPAL outcomes involved participants with valid data, and who worked full time with at least 2 days of wear time at work or worked part time with at least 1 day of wear time at work.

## Results

After emailing 71 staff members, 42 participants joined this study (response rate = 59%) (Table 1). At baseline, participants had a mean age of 38 years and mostly were female, employed full time, working in an open-plan office, university educated, and of normal BMI. Participants’ work hours were similar across the three assessment periods: mean (SD) hours worked per workday at assessments 1, 2 and 3 were 8.5 (2.2), 8.1 (2.6) and 8.3 (2.0), respectively.

**Table 1:** **Baseline characteristics for all Stand@Work participants**

**Characteristic**	**Mean (SD) or count (%)**
N	42
Sex (female)	36 (86%)
Age (years)^a^	38 (11)
Weight (kg)^b^	64 (13)
Height (cm)	165 (9)
Body mass index (kg.m^−2^)^b^	
Underweight (<18.5)	5 (13%)
Normal range (18.5 – 24.9)	20 (50%)
Overweight (25.0 – 29.9)	10 (25%)
Obese (≥30.0)	5 (13%)
Working full time	34 (81%)
Office type	
Own office	6 (14%)
Open-plan	36 (86%)
Highest level of education	
Completed all years of high school	3 (7%)
Trade, technical certificate or diploma	6 (14%)
University	33 (79%)

^a^Data missing for n = 1.

^b^Data missing for n = 2.

**Figure 2:**
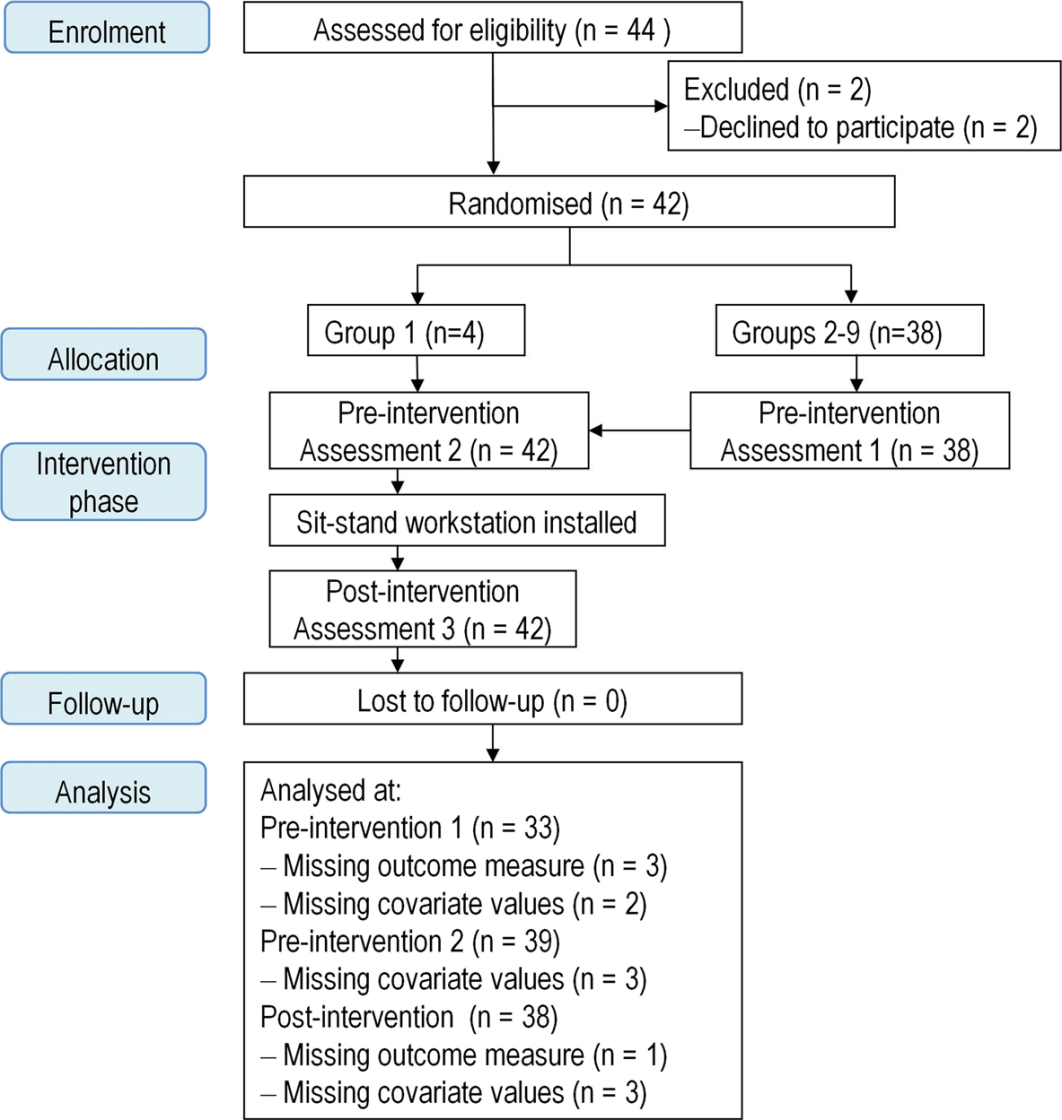
**Flow of participants in the Stand@Work study.**

Figure 2 presents the flow of participants through the study. Three participants, who were missing age or BMI values, were not included in the analyses. Imputing values for these missing covariate values did not influence the effect of the intervention on the adjusted estimates for the outcomes, nor did it change the effects age or BMI had on the outcome.

### Changes in time spent sitting at work

Table 2 presents the mean estimates for objectively assessed and self-reported time spent sitting, standing and stepping at work for the three study assessments. The first group to receive the intervention (n = 4) did not act as controls and, therefore, fewer participants were analysed at assessment 1.

**Table 2:** **Outcome measures at assessments 1, 2 and 3 for Stand@Work** ^**a**^

**Outcome (minutes per day)**	**Assessment 1**		**Assessment 2**		**Assessment 3**	
	**N**	**Mean (SD)**	**N**	**Mean (SD)**	**N**	**Mean (SD)**
Time at work spent (ActivPAL)^b^						
Sitting	32	347 (59)	36	364 (73)	36	282 (80)
Standing	32	45 (28)	36	47 (27)	36	128 (69)
Stepping	32	50 (23)	36	54 (31)	36	60 (33)
Time at work spent (OSPAQ)						
Sitting	33	403 (114)	39	374 (152)	38	271 (115)
Standing	33	48 (29)	39	52 (50)	38	156 (76)
Walking	34	56 (40)	39	56 (35)	38	67 (41)
Heavy labour	35	6 (14)	39	8 (16)	38	9 (18)
Time spent sitting on work day (WSQ)						
Transport	31	74 (54)	36	86 (62)	35	84 (53)
At work	31	426 (121)	36	393 (91)	35	305 (121)
Watching TV	31	86 (57)	36	121 (75)	35	95 (46)
Using computer at home	31	57 (66)	36	50 (47)	35	66 (77)
Other leisure activities	31	70 (74)	36	64 (58)	35	77 (60)
Total sitting time	31	712 (161)	36	714 (160)	35	627 (180)
Time spent sitting on non-work day (WSQ)						
Transport	31	68 (60)	36	72 (54)	35	71 (69)
At work	31	41 (103)	36	32 (96)	35	22 (61)
Watching TV	31	128 (75)	36	165 (96)	35	124 (71)
Using computer at home	31	105 (107)	36	91 (74)	35	90 (99)
Other leisure activities	30	240 (165)	36	196 (103)	35	224 (121)
Total sitting time	30	588 (266)	36	557 (187)	35	531 (215)

^a^Excludes participants with missing covariate values (BMI and age).

^b^ActivPAL analyses involved participants with at least 75% wear time at work, and who worked fulltime with at least 2 days of wear time at work or worked part time with at least 1 day of wear time at work.

Table 3 shows the estimates of change in outcomes for the control and intervention conditions after adjusting for study group, measurement time and a range of covariates. The analyses for the control condition tested for differences between assessments 1 and 2 (assessment 2 minus 1) and the intervention analyses tested for differences between assessments 2 and 3 (assessment 3 minus 2). In the intervention condition, we observed statistically significant changes in participants’ objectively assessed and self-reported sitting and standing during work. Both objectively measured and self-reported sitting time at work decreased while standing time increased. In the control condition, we did not find statistical differences in participants sitting or standing during work by objective or self-reported measures. There were no changes in time spent stepping at work in both intervention and control conditions. The differences in change in objective time spent sitting and standing at work between the intervention and control conditions were significant (p = 0.004 and p < 0.001, respectively), while the intervention vs. control comparison was only significant for self-reported standing time at work (p < 0.001) and not for sitting time at work (p = 0.075).

**Table 3:** **Adjusted estimates of change in outcomes for control and intervention conditions for Stand@Work** ^a^

**Outcome (minutes per day)**	**Control (Assessment 2 minus 1)** ^**b**^			**Intervention (Assessment 3 minus 2)** ^**c**^			**Control vs. Intervention** ^**d**^
	**Estimate**	**(95% CI)**	**p-value**	**Estimate**	**(95% CI)**	**p-value**	**p-value**
Time at work spent (ActivPAL)^e^							
Sitting	10	(−24, 43)	0.565	−73	(−106, −39)	<0.001	0.004
Standing	4	(−14, 21)	0.678	65	(47, 83)	<0.001	<0.001
Stepping	3	(−9, 16)	0.596	11	(−1, 24)	0.081	0.453
Time at work spent (OSPAQ)							
Sitting	−14	(−66, 37)	0.576	−91	(−142, −40)	0.001	0.075
Standing	11	(−15, 36)	0.401	99	(74, 125)	<0.001	<0.001
Walking	2	(−15, 19)	0.823	13	(−4, 30)	0.127	0.433
In heavy labour at work	3	(−2, 7)	0.211	0	(−4, 5)	0.927	0.454
Time spent sitting over a whole workday (WSQ)							
Transport	14	(−5, 34)	0.138	2	(−17, 21)	0.826	0.424
At work	−37	(−86, 11)	0.130	−74	(−123, −25)	0.004	0.365
Watching TV	30	(6, 55)	0.016	−26	(−51, −2)	0.038	0.007
Using computer at home	−3	(−22, 16)	0.788	14	(−5, 34)	0.141	0.256
Other leisure activities	−2	(−34, 29)	0.875	5	(−26, 37)	0.731	0.763
Total sitting time	5	(−70, 80)	0.895	−80	(−155, −4)	0.040	0.179
Time spent sitting over a whole non-workday (WSQ)							
Transport	11	(−16, 38)	0.437	7	(−20, 34)	0.604	0.876
At work	−3	(−42, 37)	0.891	−5	(−45, 35)	0.807	0.948
Watching TV	32	(−7, 71)	0.102	−46	(−86, −7)	0.021	0.019
Using computer at home	−20	(−54, 14)	0.247	−6	(−41, 28)	0.716	0.621
Other leisure activities	−43	(−109, 24)	0.203	11	(−56, 77)	0.746	0.338
Total sitting time	−29	(−130, 72)	0.567	−40	(−142, 61)	0.432	0.895

^a^Estimates adjusted for measurement time, study group, sex, age, BMI, office type, education level, and full time or part time work.

^b^The control analyses tested the difference between assessments 1 and 2 (assessment 2 minus assessment 1).

^c^The intervention analyses tested the difference between assessments 2 and 3 (assessment 3 minus assessment 2).

^d^An F test was used to compare estimates between control and intervention conditions for each outcome.

^e^ActivPAL analyses involved participants with at least 75% wear time at work, and who worked full time with at least 2 days of wear time at work or worked part time with at least 1 day of wear time at work.

Initial analyses with only adjustment for study group and measurement time (Additional file 1: Table S1) produced the same pattern of results, except objectively measured stepping time at work increased significantly in the intervention condition (estimate, 13 min/day (95%CI: 1, 25), p = 0.035) but not in the control condition (estimate, 3 min/day (95%CI: −9,15), p = 0.645); however, the comparison between intervention and control was not significant (p = 0.316).

### Changes in time spent sitting over the whole day

For self-reported sitting time on a work day, we found significant reductions in total sitting time in the intervention condition but not in the control condition, although the difference between the two conditions was not significant (p = 0.179) (Table 3). There were also significant changes in self-reported time spent watching TV on a work day in both groups: we observed an increase in workday TV-viewing in the control condition and a reduction in workday TV-viewing in the intervention condition (between condition comparison, p = 0.007). Time spent watching TV on a non-workday was also significantly different between the intervention and control conditions (p = 0.019) with a decrease observed in the intervention condition and no change found in the control condition. There were no changes in self-reported sitting time in other domains on workdays or non-workdays. Preliminary analyses only adjusting for study group and measurement time yielded the same pattern of results (Additional file 1: Table S1).

## Discussion

The Stand@Work pilot study adds to the current literature around reducing sitting time at work and promoting workplace wellness with evidence generated using a randomized controlled design in a resource-limited context. This study has shown that modifying the existing workplace environment by installing a sit-stand workstation is effective for reducing desk-based workers’ sitting time at work, which also resulted in a similar reduction in total sitting time over the whole day in the short term. The reductions in sitting time were mainly accounted for by increases in time spent standing.

In Stand@Work, sitting time at work decreased in the intervention condition compared to the control condition (73 min/day and 74–91 min/day when measured objectively and by self-report, respectively). These results are consistent with those from previous sit-stand workstation intervention studies which found that sitting time at work significantly decreased in the sit-stand workstation intervention versus a comparison condition: on average, reductions in work time sitting of 66 min/day by self-report [26], and ranged from 33 min/day to 143 min/day by objective measurement [23,25,27]. Our results are similar to those reported in the Neuhaus et al. RCT [27], which found that sitting time (measured by ActivPAL) reduced by 33 and 89 min/8-hr workday in a sit-stand workstation only condition and a multicomponent condition involving organisation and individual level strategies, respectively, relative to the comparison group at three months follow-up. The mean change in objectively measured time spent sitting at work was about one hour less in this study compared to those reported in earlier sit-stand desk comparison studies [23,25]. This may be explained by differences in intervention design and study sample: the intervention by Healy and colleagues had additional intervention components complementing the introduction of a sit-stand workstation [25], while the Alkhajah et al. study participants consisted of sedentary behaviour researchers as intervention group participants which might have been a more motivated group [23].

Total sitting time over a workday declined in Stand@Work participants during the intervention according to self-report. These results are partially consistent with those from one intervention study indicating that objectively assessed sitting time decreased by over an hour on a whole day after using sit-stand workstations for one week and three months [23]. Other sit-stand workstation trials did not examine their effects on sitting time over the whole day [24-26].

Based on self-reported time spent sitting in specific domains, the reduction in whole day sitting time in Stand@Work appeared to be partly a result of a decrease in TV-viewing in the intervention compared to the control condition. This reduction in TV-viewing is not easy to explain. It is unlikely that this was caused by variations in weather or television programming, as the intervention and control assessments took place simultaneously and measurement time was accounted for in the analyses. One might expect workers to compensate for less sitting at work with more sitting in other non-work domains [36], although compensation for occupational sitting with less sitting in leisure time has not been found in previous studies [9,12,14]. Less time watching TV would be a welcome side effect of the intervention, as the average adult spends 85-90% of their leisure time sedentary [37,38], leaving large potential for public health improvements. Before much value is placed on this finding, future studies should replicate this result.

Reducing desk-based workers’ sitting time and increasing their activity time at work are likely to have cumulative physiological benefits over the course of a day [39,40]. In Stand@Work, participants reported sitting almost 12 hours over a whole workday (close to 6 h/day at work) pre-intervention, and reduced their sitting time, total and at work, by well over an hour in the intervention condition. Epidemiological evidence suggests that every hour of daily sitting time is associated with a 2% increase in all-cause mortality, and that the non-linear risk association increases to 5% for adults sitting >7 h/day [2]. Additionally, even small changes in walking or stepping could be clinically meaningful. For example, Stand@Work participants increased their stepping time by 11 min/day in the intervention condition, although this was not statistically significant. Laboratory evidence shows that breaking up prolonged periods of sitting (5–9 hours) with short walking breaks of light or moderate-intensity (84 sec – 2 min) improved blood glucose and insulin levels in adults [41,42].

While the exact amount of sitting and light activity that is significant to health remains to be determined [1], it is important that researchers develop, test and refine strategies to reduce sitting time at work, such as sit-stand workstations and other occupational sitting reduction approaches, including computer prompted standing and activity breaks [43,44]. Different approaches may have different effects on workers in a variety of desk-based work settings, additionally issues around the acceptability of sitting and standing to work as well as perceptions of choice (e.g., forced standing vs. optional standing) should be investigated. The sustainability and suitability of different approaches with respect to workplace design, employer costs and organizational support also remain to be established.

An encouraging finding of this study was that the participants substantially reduced their sitting time during work by simply providing them with a sit-stand workstation and no additional support to actually use it. It seems that adding additional intervention components that include behavioural change techniques (e.g. self monitoring, goal setting, prompting, rewarding, etc.) might yield additional benefits. The studies by Neuhaus et al. [27] and Healy et al. [25] showed larger reductions in sitting time after supplementing the sit-stand workstations with organisational and individual-level intervention components.

### Strengths and limitations

The strengths of this study include having a randomized controlled cross over design, using objective measurement of sitting and standing, and having strong staff and management support for evaluation. The novel study design made use of limited resources to inform organisational decision making related to workplace wellness practices. Limitations include short term follow-up and a lack of blinding to study allocation, which was not possible due to the open plan office setup. Additional follow up would have strengthened results, but this was not possible as participants could only trial the sit-stand workstations for four weeks and we did not have the opportunity to re-assess their behaviours at 12 months. Furthermore, Stand@Work involved a convenience sample of participants working in a health-related field who were mostly female and had tertiary education levels, similar to previous sit-stand desk intervention studies [23,25-27]. Future exploration is needed to see whether these results would be found in other office/desk-based worker groups, such as those with non-tertiary education levels, in conjunction with formative work to determine the feasibility and acceptability of the sit-stand workstation as a strategy for other non-health related workplaces. For example, it has been reported that uptake of sit-stand workstation use varied depending on whether the devices were electronically or manually operated [28], while the ‘hot’ desk strategy only had partial uptake [24], even though people who used the sit-stand ‘hot’ desk only used it in the standing position.

Given the increasing awareness about the potential health risks from extended occupational sitting, and the substantial focus on workplace wellness, these study findings can potentially inform employer decisions about environmental approaches to reducing their employees’ sitting time at work. While the evidence on sitting, health and occupational outcomes is not yet sufficient to develop a business case for wide scale investment in sit-stand workstations; specifically, there is a need for ongoing intervention evidence, conducted in a variety of office settings, to inform current policy and practice. Up front cost is a likely barrier for many workplaces to roll out this type of intervention on a larger scale, so demonstrating longer term effectiveness is important. Evaluating other lower-cost behavioural strategies aimed at reducing extended sitting at work is also warranted, to determine whether similar reductions in sitting time can be achieved. Investigating the effects of changing sitting behaviour on work-related outcomes, such as productivity, is required as well. From a policy perspective, the message to reduce occupational sitting time is additional to the existing evidence that supports ongoing investment in workplace physical activity promotion.

## Conclusion

The Stand@Work study found that unprompted introduction of sit-stand workstations in the workplace can substantially reduce sitting time in the short term. As the qualitative focus group results of the Stand@Work study have previously also shown the feasibility of sit-stand workstation usage [45], these workstations might have public and occupational health potential for an increasingly sedentary workforce. Future studies should focus on the longer term effectiveness of integrated sedentary behaviour interventions based around sit-stand workstations.

## Additional file

Additional file 1: Table S1. Partially adjusted estimates of change in outcomes for control and intervention conditions for Stand@Work.^1^
