# Supplementary material for: The effectiveness of sit-stand workstations for changing office workers’ sitting time: results from the Stand@Work randomized controlled trial pilot
Source: Int J Behav Nutr Phys Act. 2014 Oct 8;11:127. doi: 10.1186/s12966-014-0127-7 (PMC4194364; doi:10.1186/s12966-014-0127-7)
Supplement: Additional file 1: Table S1. — Partially adjusted estimates of change in outcomes for control and intervention conditions for Stand@Work.1 [file 12966_2014_127_MOESM1_ESM.docx]

Supplementary Table 1S. Partially adjusted estimates of change in outcomes for control and intervention conditions for Stand@Work. ^1^

| Outcome (minutes per day) | Control  (Assessment 2 minus 1) ^2^ | | |  | Intervention  (Assessment 3 minus 2) ^3^ | | |  | Control vs. Intervention ^4^ |
| --- | --- | --- | --- | --- | --- | --- | --- | --- | --- |
|  | Estimate | (95% CI) | p-value |  | Estimate | (95% CI) | p-value |  | p-value |
|  |  |  |  |  |  |  |  |  |  |
| Time at work spent (ActivPAL) ^5^ |  |  |  |  |  |  |  |  |  |
| Sitting (min) | 5 | (-25, 36) | 0.727 |  | -74 | (-105, -44) | 0.000 |  | 0.003 |
| Standing (min) | 4 | (-12, 21) | 0.593 |  | 68 | (52, 84) | 0.000 |  | 0.000 |
| Walking (min) | 3 | (-9, 15) | 0.645 |  | 13 | (1, 25) | 0.035 |  | 0.316 |
|  |  |  |  |  |  |  |  |  |  |
| Time at work spent (OSPAQ) |  |  |  |  |  |  |  |  |  |
| Sitting | -30 | (-79, 19) | 0.222 |  | -104 | (-151, -56) | 0.000 |  | 0.070 |
| Standing | 12 | (-12, 37) | 0.325 |  | 103 | (79, 127) | 0.000 |  | 0.000 |
| Walking | 6 | (-10, 23) | 0.447 |  | 10 | (-6, 27) | 0.225 |  | 0.792 |
| In heavy labour at work | 3 | (-1, 7) | 0.169 |  | 1 | (-3, 5) | 0.693 |  | 0.529 |
|  |  |  |  |  |  |  |  |  |  |
| Time spent sitting over a whole workday (WSQ) | | |  |  |  |  |  |  |  |
| Transport | 12 | (-6, 30) | 0.187 |  | -3 | (-21, 15) | 0.737 |  | 0.306 |
| At work | -44 | (-89, 1) | 0.056 |  | -82 | (-127, -37) | 0.001 |  | 0.322 |
| Watching TV | 26 | (4, 49) | 0.022 |  | -27 | (-50, -5) | 0.018 |  | 0.006 |
| Using computer at home | -5 | (-23, 13) | 0.567 |  | 13 | (-5, 30) | 0.160 |  | 0.209 |
| Other leisure activities | -2 | (-31, 28) | 0.900 |  | 5 | (-24, 35) | 0.715 |  | 0.772 |
| Total sitting time | -11 | (-82, 60) | 0.755 |  | -96 | (-168, -25) | 0.009 |  | 0.157 |
|  |  |  |  |  |  |  |  |  |  |
| Time spent sitting over a whole non-workday (WSQ) | | |  |  |  |  |  |  |  |
| Transport | 14 | (-12, 40) | 0.273 |  | 3 | (-23, 29) | 0.839 |  | 0.592 |
| At work | -1 | (-40, 38) | 0.959 |  | -1 | (-40, 37) | 0.947 |  | 0.993 |
| Watching TV | 28 | (-10, 65) | 0.142 |  | -44 | (-81, -6) | 0.022 |  | 0.027 |
| Using computer at home | -17 | (-49, 15) | 0.288 |  | -2 | (-33, 30) | 0.917 |  | 0.557 |
| Other leisure activities | -40 | (-102, 23) | 0.209 |  | 14 | (-48, 76) | 0.647 |  | 0.311 |
| Total sitting time | -21 | (-114, 72) | 0.650 |  | -33 | (-126, 59) | 0.476 |  | 0.877 |
|  |  |  |  |  |  |  |  |  |  |

1. Estimates adjusted for measurement time and study group only.
2. The control analyses tested the difference between assessments 1 and 2 (assessment 2 minus assessment 1).
3. The intervention analyses tested the difference between assessments 2 and 3 (assessment 3 minus assessment 2).
4. An F test was used to compare estimates between control and intervention conditions for each outcome.
5. ActivPAL analyses involved participants with at least 75% wear time at work, and who worked full time with at least 2 days of wear time at work or worked part time with at least 1 day of wear time at work.
